# A Review on Lactoferrin and Central Nervous System Diseases

**DOI:** 10.3390/cells10071810

**Published:** 2021-07-17

**Authors:** Yu-Qi Li, Chuang Guo

**Affiliations:** College of Life and Health Sciences, Northeastern University, No. 195, Chuangxin Road, Hunnan District, Shenyang 110169, China; 20185666@stu.neu.edu.cn

**Keywords:** lactoferrin, iron homeostasis, central nervous system diseases, neurodevelopment, neuroprotection

## Abstract

Central nervous system (CNS) diseases are currently one of the major health issues around the world. Most CNS disorders are characterized by high oxidative stress levels and intense inflammatory responses in affected tissues. Lactoferrin (Lf), a multifunctional iron-binding glycoprotein, plays a significant role in anti-inflammatory, antibacterial, antiviral, reactive oxygen species (ROS) modulator, antitumor immunity, and anti-apoptotic processes. Previous studies have shown that Lf is abnormally expressed in a variety of neurological diseases, especially neurodegenerative diseases. Recently, the promotion of neurodevelopment and neuroprotection by Lf has attracted widespread attention, and Lf could be exploited both as an active therapeutic agent and drug nanocarrier. However, our understanding of the roles of Lf proteins in the initiation or progression of CNS diseases is limited, especially the roles of Lf in regulating neurogenesis. This review highlights recent advances in the understanding of the major pharmacological effects of Lf in CNS diseases, including neurodegenerative diseases, cerebrovascular disease, developmental delays in children, and brain tumors.

## 1. Introduction

Lactoferrin (Lf) was first identified in bovine milk by Sorensen in 1939, and Lf was subsequently isolated from human milk in 1960 [1,2]. Lf is an iron-binding glycoprotein with a molecular weight of approximately 80 kDa. The amino acid sequence of Lf has high homology with that of transferrin (Tf), so it is classified as a member of the Tf family [3]. The secondary structure of Lf mainly consists of α-helices and β-sheets, and there are fewer β-sheets than α-helices. The polypeptide chain of Lf is composed of two highly homologous globular leaves formed by 703 amino acids, and these structures are called the N-lobe and C-lobe [4]. Each lobe, composed of two domains (N1 and N2, C1 and C2), can bind to a ferric ion, and Lf can assume both closed iron-bound (holo) and open iron-free (apo) conformational states based on its iron content [5,6,7,8]. Lf has three subtypes: α, β, and γ. The α subtype can bind to iron and has no ribonuclease activity; the other two have RNase activity but do not exhibit iron-binding [9,10]. Lf is widely distributed in various tissues and secretions of mammals, such as milk, tears, saliva, semen, nasal secretions, bronchial secretions, bile, gastrointestinal fluids and other mucosal secretions [11,12]. In addition, Lf is a monomeric glycoprotein with bactericidal activity found in neutrophil granules [13].

Lf is a multifunctional protein. In addition to its well-known function of regulating iron homeostasis [14], Lf also exerts multiple important biological effects, such as anti-inflammatory, antibacterial, reactive oxygen species (ROS) modulator, antiviral, and antitumor immunity effects [1,2,4,5,12,15,16]. These activities are largely mediated by the capacity of Lf to bind iron [7,17,18,19] and the binding constant [20,21,22]. However, the potential molecular mechanisms by which Lf exerts its multiple effects are still under investigation, and interaction of Lf with its cell receptors seems to be the most reasonable mechanism. Specific Lf-receptors (LfRs) are present in different cells of different species including the brush-border membranes of intestines and choroid plexus [20,21]. Several Lf receptors (LfRs) were discovered in different tissues and cell types and include asialoglycoprotein receptor, intelectin-1, low-density lipoprotein receptor-related protein 1 (LRP1), nucleolin, and omentin-1 [7,19]. Notably, the properties of Lf are not limited to its receptor-mediated responses, and Lf may be targeted to the nucleus, bind to specific DNA sequences, and act as a transcription factor [23,24]. Subsequent studies showed that increased levels of Lf in the body can up-regulate the expression of a variety of genes, such as genes related to the innate immune system (pathogen recognition and defense) [25], lipid metabolism (fatty acid β oxidation, fatty acid elongation, fatty acid synthesis and degradation) [26,27,28], and heterogeneous metabolism and lysosomal degradation [29,30]. As a cytokine, Lf can promote cell proliferation and differentiation in an LRP-dependent or independent manner [7,31,32,33]. A study indicated that Lf contributes to immune function by inducing the transforming growth factor β (TGF-β) and Wnt signaling pathways and the expression of genes related to innate immunity [25]. In addition, Lf can also promote cell apoptosis by regulating the levels of signaling molecules, such as caspase-3, poly(ADP-ribose) polymerase (PARP), B-cell lymphoma-2 (Bcl-2) and Bcl-2 associated X (Bax) [34]. After exogenous supplementation with Lf, the expression levels of the autophagy markers autophagy-related gene 7 (Atg7) and Atg12-Atg5 and the ratio of microtubule-associated protein 1 light chain 3 (LC3)-II/LC3-I were increased [35], suggesting the promotion of autophagy. Interestingly, ferroptosis, a novel non-apoptotic regulated form of iron- and lipid peroxidation-dependent form of cell death, may also be involved in Lf in various cancer cells [36,37]. More recently, as a broad-spectrum antiviral agent, Lf has strong immunomodulatory and anti-inflammatory properties and therefore may be a potential treatment for coronavirus disease 2019 (COVID-19) [38,39].

In fact, research continues to characterize novel pleiotropic biological roles for Lf; Lf was observed in the human brain by immunohistochemistry and was associated with aging, particularly with multiple central nervous system (CNS) diseases, including Alzheimer’s disease (AD), Parkinson’s disease (PD), Down’s syndrome, Pick’s disease, sporadic amyotrophic lateral sclerosis, amyotrophic lateral sclerosis, cerebrovascular disease, developmental delay in children and other neurological diseases [35,40,41,42,43,44,45,46,47,48]. Evidence suggests that CNS diseases usually cause a strong inflammatory response, and their pathogenesis is also closely related to neuroinflammation and oxidative stress [46,49,50,51]. Thus, it can be hypothesized that Lf exerts various beneficial health effects in CNS diseases, at least by improving immune system function and ROS modulator capacity [52]. However, our understanding of the roles of Lf proteins in the initiation or progression of CNS diseases is limited, especially the roles of Lf in regulating neurogenesis, such as neuronal cell proliferation, differentiation, migration and synaptic connections [45]. 

In this review, we summarized the current knowledge, presented a better understanding of the functional role of Lf in CNS diseases, and tried to provide a theoretical basis for its potential future use in preventive and therapeutic applications.

## 2. Lf in Neurodegenerative Diseases

### 2.1. The Important Role of Lf in the Onset and Prevention of Neurodegenerative Diseases

Neurodegenerative diseases are diseases caused by the loss of cells and neurons in the brain or spinal cord, and these diseases may lead to cognitive and behavioral impairment and even death in severe cases [51]. AD and PD are relatively well-known neurodegenerative diseases. The common mechanisms underlying the pathogenesis of neurodegenerative diseases mainly include oxidative stress, neuroinflammation, iron and other transition metal disorders, lipid metabolism imbalance, etc., [51,53,54]. As mentioned above, Lf was found in the lesion areas of a variety of neurodegenerative diseases, indicating that it is closely related to the pathogenesis of neurodegenerative diseases. Due to the presence of LfR on brain capillary endothelial cells and neurons, exogenous Lf can easily cross the blood-brain barrier (BBB) and reach neural tissues; thus, Lf was successfully used as a potential brain-targeting delivery system [55,56,57,58,59]. Increasing numbers of studies indirectly suggest that the Lf-mediated neuroprotection against neurodegenerative disorders is attributed to its biological characteristics. (1) In inflamed tissues, the content of Lf is significantly increased [60], and Lf can reduce the expression of the pro-inflammatory cytokines tumor necrosis factor (TNF)-α, interleukin (IL)-1β, and IL-6 to exert its anti-inflammatory effects [61]; (2) Lf can significantly increase the content of superoxide dismutase (SOD) and reduce the generation of ROS by inhibiting the Fenton reaction [16], thereby exerting ROS modulator effects; (3) Lf can regulate the expression of proteins involved in lipid degradation by controlling the activity of the cAMP/extracellular regulatory protein kinase (ERK) signaling pathway via LRP1 [28], thereby regulating lipid metabolism; (4) increased LfR expression in the brains of patients with neurodegenerative diseases suggests that Lf may play a significant role in the pathogenesis and prevention of neurodegenerative diseases [62]; (5) Lf acts as a normoxic mimetic of hypoxia that is capable of stabilizing hypoxia-inducible factor 1α (HIF-1α) [63] (6); Lf elevates the mRNA and protein levels of brain-derived neurotrophic factor (BDNF) and components of its signaling pathway [64]. In addition to the abovementioned mechanisms, other potential molecular mechanisms are still under investigation.

### 2.2. Alzheimer’s Disease

AD, a neurodegenerative disease, is the most common form of dementia affecting elderly people. Accumulating evidence indicates that the pathology of AD is characterized by abnormal levels of extracellular senile plaques (SPs) containing insoluble amyloid-β (Aβ) peptides, abnormal levels of intracellular neurofibrillary tangles (NFTs) containing hyperphosphorylated tau [65,66,67], and abnormal iron deposition and oxidative damage [68,69,70] in the brains of patients. The main mechanism underlying AD is that β-amyloid precursor protein (APP) produces significant levels of neurotoxic Aβ42 through the amyloid degradation pathway; then, neurotoxic Aβ42 can induce tau protein aggregation and hyperphosphorylation, and hyperphosphorylated tau and normal tau compete for binding to tubulin, disrupting the dynamic balance of microtubule assembly and decomposition, and ultimately form NFTs [71], causing region-specific synaptic degeneration and neuronal loss and leading to dementia [72]. In addition to the above pathways, AD can also be triggered by neuroinflammatory pathways [73]. There are a large number of activated astrocytes and microglia in the brains of AD patients, accompanied by increased expression of TNF-α, IL-1β, IL-6 and other inflammatory factors [74,75].

The levels of Lf, which acts both as an iron-binding protein and inflammatory modulator, are markedly increased in the brains of patients with AD [41,44,76,77]. Further studies confirmed that a large amount of naturally Lf is deposited in the areas of AD patient brains where SPs and NFTs are enriched [41,44,78]. Although the precise origin of Lf in AD remains unknown, a previous study suggested that the Lf protein is mainly synthesized in the brain by reactive microglia and/or infiltrating monocytes/macrophages [77], suggesting an endogenous neuroprotective mechanism. Of course, the possibility that some Lf protein is transported from peripheral circulation into the brain cannot be excluded [55,58,59,76,79]. The role of Lf in the brains of patients with AD remains to be further elucidated, but inhibiting the inflammatory response is at least one of its important functions. Previous studies raise several possible explanations for this effect. Lf may directly suppress the secretion of inflammatory cytokines [11,19,80,81] and inhibit complement C3 activation and deposition [82,83], thus suppressing the classical complement pathway in the brains of patients with AD [84]. Another possibility is that Lf has antioxidant properties because oxidative stress is tightly associated with the pathology of AD [85], and increased oxidative damage is an early event in AD pathogenesis [86]. Interestingly, redox-active iron, a source of redox-generated free radicals that are conducive to oxidative damage, is associated with the SPs and NFTs in AD [70]; however, Lf tightly binds to iron and thus has ROS modulator properties [2].

In fact, the ROS modulator and anti-inflammatory effects of Lf may serve as a possible protective mechanism against AD by modulating the phosphorylated protein kinase B (p-PKB/AKT)/phosphatase and tensin homolog (PTEN) pathway; this possibility was investigated and confirmed in the AD patients [87]. In addition, for the Aβ plaques that are generated, supplementation with Lf can degrade the oxidative metabolism of a variety of exogenous and endogenous substances by upregulating the expression of cytochrome P450 enzymes and other related proteins [52]. Nevertheless, we hypothesize that the function of Lf may be similar to that of an iron chelator, which can induce the expression of HIF-1α, thereby exerting neuroprotective effects [88,89]. Based on this, we conducted a series of studies and found that Lf can promote the non-amyloid metabolism of APP by activating disintegrin and metalloproteinase 10 (ADAM10) [88]. Soluble APPα (sAPPα), whose expression is induced by Lf, can significantly inhibit the formation of Aβ plaques in the cerebral cortex and hippocampus and improve spatial cognition and learning ability. Moreover, it was confirmed that Lf induced the upregulation of ADAM10 by regulating the ERK1/2-cAMP response element-binding protein (CREB) and HIF-1α signaling pathways [88]. These studies suggest that exogenous Lf can improve cognitive decline, making it tempting to speculate that the increased Lf levels in the lesion areas of patients with AD may be a defensive response of the brain. 

In recent years, several studies have explored the role of salivary Lf as a potential biomarker of AD [90,91], and the expression of Lf in the brain continues to increase with age [92], supporting the possible use of salivary Lf levels in the differential diagnosis of AD. 

### 2.3. Parkinson’s Disease

Parkinson’s disease (PD), also known as “trembling paralysis”, is a common neurodegenerative disease whose incidence rate is only inferior to that of AD. Clinically, PD is mainly characterized by resting tremor, stiffness, and retardation; however, PD patients usually have non-motor symptoms, such as dysosmia, sleep disorder, affective disorder, autonomic nervous dysfunction, anxiety, depression and cognitive impairment, before the onset of dyskinesia. The neuropathological features of the disease are the death of dopaminergic neurons in the substantia nigra (SN) and the formation of Lewy bodies (LBs) by α-syn deposition. Although the details of the mechanism underlying the development of PD have not yet been elucidated, iron accumulation, increased inflammation and oxidative stress, and lipid peroxidation damage are also considered to be significant pathological features of PD [93]. A large number of studies have suggested that there are observable iron deposits in the SN area in PD patients and that iron deposits may occur earlier than the onset of Parkinson’s symptoms [94,95,96]. Iron overload may cause damage to dopaminergic neurons by activating microglia [97,98,99].

Early studies have found that the expression of naturally Lf is significantly increased in the remaining midbrain neurons of PD patients [43]. Combined with the high expression of the Lf receptor in these neurons and in vascular endothelial cells [62], it is hypothesized that circulating Lf in the peripheral blood enters the brain through receptor-mediated one-way transcytosis and reaches PD-prone brain regions [55]. The function of circulating Lf in the brain may be to protect these susceptible neurons rather than to cause iron accumulation and neuronal death [100]. Regarding the neuroprotective effect of naturally Lf in PD, the exogenous Lf has been widely recognized [30,89,100,101,102,103,104]. Moreover, studies have shown that Lf inhibits MPTP-induced excessive iron accumulation by upregulating the main iron regulatory proteins, divalent metal transporter (DMT1) and transferrin receptor (TFR), in cells [89,99]. However, in vitro, holo-Lf and apo-Lf exhibit similar behavior, so the neuroprotective effect of Lf is not limited to its iron chelation [100]. In addition to protecting dopaminergic neurons by affecting iron metabolism, Lf enhances the expression of BDNF through the ERK-CREB pathway and HIF-1α-dependent mechanisms to reverse the movement disorders caused by PD [64,89,103]. A recent study showed that Lf may inhibit the Bcl-2/bax-mediated mitochondrial apoptosis pathway and the caspase protease family-mediated Fas/FasL exogenous apoptotic pathway [30]. Furthermore, Lf pretreatment partially reversed the decrease in mitochondrial potential, suggesting that Lf can protect cells from MPP^+^-induced oxidative stress by restoring mitochondrial function [30,104].

However, there are still many questions to be answered regarding the increase in the naturally Lf levels in the PD brain. First, the expression of Lf was detected in neurons, microglia and oligodendrocytes in PD patients [43]; however, there was no significant difference in the level of Lf in the plasma/serum or even in the cerebrospinal fluid (CSF) between PD patients and controls [105]. A case-control study further suggested the existence of endogenous Lf. This study found that transferrin gene polymorphisms (including Lf polymorphisms) exist in the brains of PD patients; that is, genetic variation in Lf can affect individual susceptibility to PD and increase the risk of PD [106]. In fact, it was reported that Lf can be synthesized in situ in the brain [107,108,109]. Although the levels of *lactoferrin* mRNA detected in the brain tissues of adult mice are very low compared with those detected in lactating mammary glands, MPTP can induce an increase in both the RNA and protein levels of Lf [109]. Subsequent studies suggested that endogenous Lf is only secreted by activated microglia [107], which is still widely accepted [100]. The function of Lf synthesized in situ in the pathological progression of PD remains to be elucidated.

### 2.4. Prion Disease

Prion diseases are fatal neurodegenerative diseases caused by the transformation of prion proteins in normal cells into subtypes of amyloidosis through conformational changes. Early studies showed that Lf causes PrP(C) to remain on the cell surface by reducing its internalization and interacts with PrP(C) and PrP(Sc) to mediate changes in their conformation; thus, Lf has functional anti-prion activity [110]. The synthetic peptide PrP (106–126) is commonly used to explore the molecular mechanism underlying prion disorders [111]. Several studies have demonstrated that Lf treatment may prevent PrP (106–126)-induced neuronal cell death by reducing ROS generation, decreasing mitochondrial dysfunction, and ensuring HIF-1α stability through the inhibition of the enzymatic activity of prolyl hydroxylase 2 (PHD2) [112,113]. 

These findings suggest that Lf may have clinical benefits in patients with neurodegenerative disorders, including AD, PD, and prion disease.

## 3. Lactoferrin in Cerebrovascular Disease

Cerebrovascular disease refers to all kinds of cerebral vascular diseases, including both large and small vessel diseases, such as cerebral atherosclerosis, thrombosis, stenosis, occlusion, cerebral arteritis, cerebral artery injury, cerebral aneurysm, intracranial vascular malformation, and cerebral arteriovenous fistula. The consequences of these diseases are among the leading health issues worldwide, and their common features are cerebral ischemia, hemorrhagic accidents and other forms of neurological dysfunction and degeneration [114]. Thus, the implementation of effective strategies for preventing cerebrovascular diseases is of utmost importance to reduce the occurrence of these diseases in an aging population. Epidemiological, pathological, clinical, and experimental evidence suggests that dietary strategies remain crucial for improving cardiovascular disease and stroke [115]. Among dietary ingredients, the increased intake of dietary protein and milk (especially whey protein) is associated with decreased risk of stroke [116,117]. Recently, Lf, a component of whey protein, was shown to play a key role in facilitating weight loss and improving the neurological deficits associated with stroke [118,119]. The underlying mechanisms could include the inhibition of hypoperfusion, hypoxia, and neuroinflammation.

### 3.1. Lf Protects against Cerebral Ischemia and Hypoxia by Interfering with Cellular Proinflammatory Factors

Stroke, also known as cerebral apoplexy or cerebrovascular accident, is always accompanied by symptoms of ischemia or hemorrhagic injury, which are the main clinical manifestations in brain tissues, and is associated with high mortality and disability rates. Stroke can be mainly divided into hemorrhagic stroke (cerebral hemorrhage or subarachnoid hemorrhage) and ischemic stroke (cerebral infarction, cerebral thrombosis), and cerebral infarction is the most common type of stroke. At present, there is still a lack of effective treatment measures.

Accumulating evidence shows the protective effect of Lf in ischemia-reperfusion injury of the heart [120,121], aorta [122], liver [123,124], gut [125,126], and lung [127], and this effect is exerted via its anti-oxidative, anti-inflammatory, and anti-apoptotic activities. Supplementation with exogenous Lf promotes angiogenesis in response to ischemia via an Src-Akt-eNOS–dependent pathway [128]. In the brain, Lf was utilized to transport nanoparticles across the BBB and achieve specific effects in protecting the brain from ischemia-reperfusion injury [129]. Neuroprotective effects of Lf on immature brains were demonstrated in rat models of cerebral ischemia hypoxia [35]. Lf significantly reduced the activation of TNFα and IL-6 gene transcription and increased the levels of p-AKT [35]. Another study demonstrated that Lf could inhibit the expression of Toll-like receptor 4 (TLR-4) and downstream inflammatory proteins, including nuclear factor-κB (NF-κB), TNF-α, and IL-1β, triggered by anoxia and reoxygenation and ischemic reperfusion [130]. In addition to its anti-inflammatory effects, it is believed that Lf can activate the Keap1/nuclear factor erythropoietin-2-related factor 2 (Nrf2) signaling pathway in ischemic brains and stimulate Nrf2 translocation from the cytoplasm to the nucleus by upregulating erythropoietin (EPO) synthesis [131]. Lf is transferred to the nucleus and binds to antioxidant response elements (AREs) in DNA, acting as a transcription factor to maintain the balance of cellular redox reactions [132,133]. As a regulator, Nrf2 also mediates the expression of many genes encoding cytoprotective, antioxidant, and anti-inflammatory proteins, such as *heme oxygenase 1 (HO-1), peroxidase,* *glutamate cysteine ligase, and glutathione peroxidase*, and can directly participate in the protection of brain cells from the damage associated with ischemic stroke [134].

### 3.2. Effect of Lf on Ameliorating Cerebral Hemorrhage

Cerebral hemorrhage (ICH), a subtype of stroke, is a destructive neurological disease that damages the brain through the mechanical force of blood extravasation and the toxicity of blood components in the parenchyma, including hemoglobin/iron and neutrophils (PMNs), which may cause secondary injury after ICH [135]. However, infiltrating PMNs may release Lf to contribute to hematoma detoxification by neutralizing toxic iron and heme [136]. Studies have shown that the expression of the immunoregulatory cytokine IL-27 is upregulated by activated microglia after ICH, leading to increased production of Lf by PMNs [137]. In other words, Lf, as an iron chelator and anti-inflammatory factor, may help detoxify hematomas and relieve the destruction of brain tissue cells caused by cerebral hemorrhage [136]. Moreover, administration of exogenous Lf may also improve the neurological deficits caused by ICH [136,137]. In fact, an early clinical study found that patients with infarction without signs of bleeding or with cerebrovascular lesions undetectable by computed tomography also exhibited increased naturally Lf levels in their CSF [138]. Thus, it can be hypothesized that the high expression of Lf in the brain ameliorates cerebral hemorrhage.

## 4. Lf in Developmental Delay in Children

### 4.1. Lf and Neurodevelopment and Cognition

Almost all human neurons are formed at birth, and neurogenesis is greatly limited after birth. However, most of the synaptic connections between neurons are formed and gradually perfected after birth. Cognition refers to the ability to process information, which mainly includes the ability to sense, perceive, learn, and remember. Because cognition requires the support of advanced brain nerve structure, human cognitive abilities are closely related to brain neurodevelopment [139]. The role of iron in neuronal metabolism and neurotransmitter and myelin synthesis makes it very important for brain development and cognitive performance both antenatally and postnatally [140]. However, antenatal and early childhood iron supplementation programs have inconsistent effects in averting cognitive impairment [141]. There is a need to develop and successfully conduct effective nutritional and micronutrient intervention studies to address the effects of iron and trace element deficiencies on early and long-term cognitive disorders during childhood [142]. Interestingly, apo-Lf may induce the proliferation and differentiation of human intestinal epithelial cells by increasing the expression of TGFβ1, whereas holo-Lf fails to regulate TGFβ1 expression, suggesting that the iron status of Lf can modulate intestinal epithelium growth and maturation [143]. Sialic acid (Sia), a conditionally essential nutrient, plays a critical role in brain and cognitive development [144]. Lf, with its iron-binding ability, is an abundant sialylated glycoprotein in human milk [145], so its potential function in neurodevelopment and cognition has attracted increasing attention [45]. However, the molecular mechanisms by which Lf can improve neurodevelopment and cognitive function are not well understood. BDNF is a neurotrophic growth factor that is highly expressed in the hippocampus and cortex of the brain. BDNF plays an important role in the transmission and plasticity of neurons in the brain and participates in the development of nerves in the brain and the formation of cognition [45]. Chen et al. reported that Lf supplementation facilitates early neurodevelopment and cognition by activating the BDNF neurotrophic signaling pathway and increasing polysialylation in postnatal piglets [64]. When the BDNF signaling pathway is activated, it increases the phosphorylation of CREB, which can induce gene transcription and play a major role in the formation of cognition [146]. Additionally, BDNF can polymerize with polySia with a degree of polymerization of at least 12 Sia residues to form a BDNF-polySia complex, which promotes the growth and/or survival of neurons [147]. Thus, the increase in the BDNF-polySia complex levels in the brain might be considered to be the main mechanism by which Lf affects neurodevelopment and cognitive formation [45]. In fact, an extended study by Chen et al. provided new evidence supporting the regulation of BDNF signaling by exogenous Lf intervention and elucidated the molecular mechanisms underlying the concentration-dependent effect of Lf supplementation on promoting neurodevelopment and cognition in neonatal piglets [148].

Premature infants exhibit immature brain development, and innate nutrition deficiency is more likely to cause neurodysplasia or damage. Breastfeeding can provide the highest levels of Lf [149], reduce the risk of infection [2,150,151], and provide a good environment for the development of the brain and nerves in preterm infants [45]. Intrauterine growth restriction (IUGR) is not only associated with an increased risk of perinatal mortality but also compromises brain development and increases the risk of later mental and psychomotor developmental complications [152]. It was demonstrated that Lf supplementation may prevent some of the IUGR-induced sequelae in rat pups by increasing the expression of genes involved in the survival, differentiation, and growth of neurons, transport of iron, and signaling of glutamate and that Lf supplementation may promote neuronal and glial cell density and corpus callosum development [153]. In addition, Lf supplementation in food during lactation also alleviated acute and long-term LPS-induced cerebral alterations, such as ventricular enlargement, brain tissue loss and myelination defects [154].

Therefore, supplementation with exogenous Lf is proposed as a conditional nutrient to promote brain neurodevelopment, neuroprotection, and cognitive function of infants during the period of rapid brain growth [45].

### 4.2. Overexpression of Lf and Developmental Delay in Children

Gelatinous drop corneal dystrophy is an autosomal recessive genetic disease that can occur at an early age [155,156,157], and the clinical symptoms are growth delays in children [158]. Several studies have shown that compared with normal people, patients with gelatinous drop corneal dystrophy exhibit high levels of Lf gene expression in their corneal tissues, leading to the accumulation of intraepithelial Lf [159,160]. However, the relationship between the overexpression of Lf and developmental delay in children has not yet been reported.

## 5. Lf in Other Neurological Diseases

### 5.1. The Inhibitory Effect of Lf on Psychiatric Illness

There is evidence to suggest that many psychiatric illnesses are characterized by inflammation, with their treatments having potential anti-inflammatory actions [161]. Lf, a known anti-inflammatory agent, distinctly attenuated acute stress-induced anxiety- and depressive-like behavior as well as decreased expression of BDNF in young rats [162]. A recent extended study further confirmed that early life diets containing Lf for 4 weeks can indeed modify genes in neural circuits underlying emotion regulation and decrease anxiety-related behavior [103]. Consistently, it was demonstrated that Lf supplementation may clearly improve the depressive-like symptoms in a repeated forced-swim test stress mouse model [163]. We also found that Lf can substantially reduce anxiety-related behavior in the MPTP-induced mouse model of PD [89]. In a recently published study [164], Lf was found to promote neurite outgrowth of PC12 cells and suppress decreased locomotor activities by enhancing serotonin and dopamine release in the amygdala of ovariectomized model rats. In fact, the elevated serum level of Lf was found in schizophrenic patients as early as 1982 [165], but it seems that it has not been further concerned. Collectively, the data suggest that Lf has the distinct advantage of improving various psychiatric symptoms in the future.

### 5.2. Lf in Brain Tumors

Recently, the use of natural nutraceuticals to support anticancer standard therapy has received increasing attention as a promising approach due to their relative abundance, bioavailability, safety, low cost, effectiveness, and host immunocompatibility [166]. As early as the 1990s, anticancer activity was ascribed to Lf [167]. Moreover, research on the role of Lf in cancer therapy and its potential application as a drug nanocarrier in cancer therapy has attracted maximum interest in recent years [1]. Although many unsolved questions and controversies have emerged from studies, the anticancer effects of Lf might occur due to the high selectivity for cancer cells or electrostatic interactions associated with Lf receptors [168,169] and a wide range of molecular targets that regulate tumor proliferation, survival, migration, invasion, and metastasis [5]. Indeed, several molecular mechanisms underlying the anticancer activity of Lf were revealed, including the modulation of cell cycle progression, induction of apoptosis and ferroptosis, inhibition of cell migration, invasion, metastasis, and tumor-related angiogenesis [5,36,37], as well as immunomodulation [170]. Notably, Lf was also found to be an ideal carrier for enhancing the efficacy of chemotherapy, even for the treatment of brain tumors, due to its ability to cross the BBB [1,5,58]. Moreover, LfRs are overexpressed on the cellular surface of glioma [171], thus augmenting the specific uptake of Lf by cancer cells. There is sufficient evidence to support this concept, especially in gliomas [172]. Alternatively, Lf might be secreted by neoplastic astrocytes [171]. Although the particular mechanism requires further evaluation and validation, Lf, with its multiple applications, might represent a promising strategy for inhibiting brain tumors and brain metastases.

## 6. Perspectives and Challenges

As a major iron-binding protein found in milk and other body fluids, Lf exerts antibacterial, antiviral, anti-inflammatory, immune-modulatory and anticarcinogenic effects [1]. Because of its ability to cross the BBB through LfR-mediated transcytosis processes within brain capillary endothelial cells [44,56] and its relatively good biocompatibility, safety, stability and resistance to proteolysis [5], the use of exogenous Lf as a therapeutic agent or the use of drug-loaded Lf-based nanoparticles were explored for the treatment of neurodegenerative diseases, cerebrovascular diseases, childhood developmental delays, brain tumors and other neurological-related diseases [57,172,173]. In fact, several unsolved questions emerge from studies including the bioavailability of Lf after oral administration, differences in physicochemical properties of commercially available Lf (in particularly the iron content), and difficulties in the clinical application (dosage, durations, evaluation of the outcomes), although it exerts pleiotropic biological functions and is safe and low-cost as a potential therapeutic agent [5,174]. Of note, salivary Lf has potential as a biomarker for the diagnosis and monitoring of age-related neurodegenerative diseases, and its levels are, for example, decreased in AD [90,91]. Taken together, these data demonstrate that Lf may play important roles in the initiation and progression of CNS diseases.

As mentioned above, Lf can play a crucial role in the promotion of neurodevelopment and neuroprotection through its involvement in the modulation of iron homeostasis, oxidative stress, inflammatory responses, and innate immunity, as well as in the expression of HIF-1α and BDNF pathway components (Table 1).

However, the exact roles of Lf in these biological processes are still unclear. As a major receptor of Lf, LRP1, whose expression is up-regulated in various CNS diseases, plays an important role in the transport and metabolism of cholesterol- and apoE-containing lipoproteins [55]. It was suggested that circulating Lf levels are inversely related to fasting glucose and triglyceride levels but directly associated with high-density lipoprotein cholesterol levels [175]. Additionally, strategies involving Lf supplementation were found to improve metabolic diseases related to lipid accumulation [176,177]; therefore, the association between Lf and lipid metabolism in the brain requires investigation. Moreover, BDNF, which is mainly released by astrocytes, is known to contribute to several aspects of neuronal development and function, such as synaptic plasticity, neuronal survival and differentiation, neuronal process growth, and neuronal repair following injury [178]. Interestingly, BDNF expression is up-regulated after Lf treatment [45,64,88,89,148,153], suggesting that some functions of astrocytes may be activated.

In addition, Lf expression is greatly increased during neurodegenerative disorders and in the brains of elderly patients [44,78], whereas the origin of Lf within the brain under normal and disease conditions has not yet been determined. Indeed, in addition to the known infiltration of circulating Lf and its synthesis by activated microglia [55,77,107,109], we also observed that the expression of Lf in astrocytes was increased with aging and neurodegenerative disorders, suggesting an endogenous neuroprotective mechanism [78,171]. Thus, whether Lf can be produced by astrocytes should be carefully explored, and its function and origin need to be identified. Due to the lack of clear data from the existing studies, the establishment of animal models for comparison is of far-reaching significance for exploring the relevant mechanism by which Lf exerts its effects. More sophisticated investigations are required to fully understand the molecular mechanism associated with Lf, as well as its utility and applicability, to make the best clinical use of Lf for widespread therapeutic applications.

## Figures and Tables

**Table 1:** The active substance of lactoferrin and its role in CNS diseases.

Action Substance	CNS Diseases	Effects Of Lactoferrin	Ref.	Effects of Action Substance
TNFα	NDG diseases, CVD	down-regulation	[7]	↑
IL-1β	NDG diseases, CVD	down-regulation	[7]	↑
IL-6	NDG diseases, CVD	down-regulation	[7]	↑
ROS	NDG diseases	down-regulation	[13]	↑
ADAM10	NDG diseases	up-regulation	[80]	↓
DMT1	NDG diseases	up-regulation	[81,91,95]	↓
TFR	NDG diseases	up-regulation	[81,91,95]	↓
PHD2	NDG diseases	down-regulation	[105,106]	↑
HIF-1α	NDG diseases, CVD	up-regulation	[55,80,81,105,124]	↓
NF-κB	CVD	down-regulation	[123]	↑
p-AKT	CVD	up-regulation	[29]	↓
TLR-4	CVD	down-regulation	[123]	↑
BDNF	NDG diseases, Developmental delay in children, psychiatric illness	up-regulation	[37,56,80,81,141,146]	↓
Casepase-3	NDG diseases, Brain tumors	up-regulation	[28]	↓
PARP	NDG diseases, Brain tumors	up-regulation	[28]	↓
Bcl-2	NDG diseases, Brain tumors	up-regulation	[28]	↓
Bax	NDG diseases, Brain tumors	up-regulation	[28]	↓

↑, stimulatory effects; ↓, inhibitory effect; NDG, neurodegenerative; CVD, cerebrovascular disease; CNS, central nervous system.
